# The Effects of Dinner-to-Bed Time and Post-Dinner Walk on Gastric Cancer Across Different Age Groups: A Multicenter Case–Control Study in Southeast China

**DOI:** 10.1097/MD.0000000000003397

**Published:** 2016-04-22

**Authors:** Le Xu, Xi Zhang, Jun Lu, Jia-Xi Dai, Ren-Qin Lin, Fang-Xi Tian, Bing Liang, Yi-Nan Guo, Hui-Yu Luo, Ni Li, Dong-Ping Fang, Ruo-Hua Zhao, Chang-Ming Huang

**Affiliations:** From the Department of Nursing (LX, XZ, J-XD, R-QL, B-L, Y-NG); Department of Gastric Surgery, Fujian Medical University Union Hospital (JL, F-XT, C-MH); Fujian Provincial Cancer Hospital (H-YL); Fuzhou General Hospital of Nan Jing Military Command (N-L); The Affiliated Hospital of Fujian Medical University (D-PF); and FuJian Provincial People's Hospital, Fuzhou, Fujian Province, China (R-HZ).

## Abstract

Gastric cancer (GC) remains a major killer throughout the world. Despite the dramatic decrease in GC over the last century, its etiology has not yet been well characterized.

This study investigated the possible independent and combined effects of the dinner-to-bed time and post-dinner walk on the risk for GC across different age groups.

A population-based, case–control study was conducted in southeast China, including 452 patients with GC and 465 age-, race-, and gender-matched controls. A self-designed questionnaire was used to collect information on demographic characteristics, dinner-to-bed time, post-dinner walk, and other behavioral factors. Conditional logistic regression models were used to estimate the effects of the dinner-to-bed time and post-dinner walk as well as their joint effect on the risk for GC across different age groups.

Individuals with dinner-to-bed time <3 hours were more prone to have GC (*P* < 0.001), and the shorter the dinner-to-bed time was, the higher was the risk for GC (*P*_trend_ < 0.001). Post-dinner nonwalk was associated with a 2.9-fold increased risk for GC compared with post-dinner walk (adjusted odds ratio [AOR] = 2.942, 95% confidence intervals [95% CIs] = 2.072–4.179). The interaction effect of dinner-to-bed time and post-dinner walk on GC risk was detected (AOR = 1.862, 95% CIs = 1.584–3.885, synergy index [SI] = 2.654, 95% CIs = 2.27–3.912). Participants with dinner-to-bed time <3 hours who did not walk after dinner were 7.4 times likely to suffer from GC (AOR = 7.401, 95% CIs = 4.523–13.16) than those with dinner-to-bed time ≥4 hours who took such walk. The risk of GC due to dinner-to-bed time <3 hours, post-dinner nonwalk and their interaction was positively correlated with age. The strongest risk was observed among people ≥70 years old, but the effects were not significant for people ≤55 years old.

Dinner-to-bed time <3 hours and post-dinner nonwalk are independent risk factors for GC; the synergistic interaction between the 2 factors was positively related to age, which might significantly increase the risk for GC among people >55 years old.

## INTRODUCTION

Gastric cancer (GC) is the 5th most frequent cancer and the 3rd leading cause of cancer mortality worldwide, with 984,000 new cases and 841,000 deaths in 2013, Of all such deaths, more than two-thirds occurred in developing countries.^1^ China has a high incidence rate of GC; an estimated 404,000 people are diagnosed with GC each year, comprising 42% of all cases worldwide.^2^ Although notable progress has achieved in the diagnosis and treatment of GC in China over recent decades, more than 300,000 cases result in death every year.^3,4^ Moreover, approximately 80% of patients are already in advanced stages when diagnosed and therefore have a poor prognosis, because of the lack of effective treatment associated with late detection.^4,5^ Hence, it is vital to identify the risk factors associated with GC to implement effective prevention and control measures.

The development of GC is widely recognized as a multifactorial and complex process.^6^ To date, the exact etiology of GC is still unclear.^7,8^ However, multiple studies have demonstrated that environmental factors are the major causes of GC, specifically, adverse lifestyles.^9–12^ In 2005, Fujiwara et al^13^ first proposed the concept of the dinner-to-bed time, which is the period between finishing eating dinner to going to bed. These authors found that dinner-to-bed of less than 3 hours was significantly associated with an increased risk of gastro-esophageal reflux disease (GERD).^13^ Then an animal experiment has further indicated that shorter dinner-to-bed time (t < 3 hours) caused GERD.^14^ Although the relationship between the dinner-to-bed time and GERD has been confirmed, few studies have examined the association between the dinner-to-bed time and cancer. In the past, only 2 studies have provided evidence that shorter dinner-to-bed time was related to the risk for gastric cardia adenocarcinoma (GCA) and esophageal squamous cell carcinoma (ESCC).^15,16^ Meanwhile, several studies have reported that post-dinner walk attenuated the risks for GERD, ESCC, and GCA due to shorter dinner-to-bed time,^15–17^ but other epidemiological studies have shown in consistent results.^18,19^ Furthermore, the previous studies on the associations between cancer and both dinner-to-bed time and post-dinner walks are mostly single-center, small sample, less demographic data and these studies did not adjust for family cancer history or *Helicobacter pylori* infection, which are potential sources of confounding. In addition, to our knowledge, no report exists regarding the interaction between dinner-to-bed time and post-dinner walk on the risk for GC across different age groups.

We thus conducted a case–control study to assess the independent and joint effects of dinner-to-bed time and post-dinner walks on the risk for GC across different age groups in a high-risk region.

## MATERIALS AND METHODS

### Study Design and Participants

A multicenter, population-based, case–control study of people with GC was conducted in southeast China from June 1st 2014 to August 31st 2015. Participants were randomly recruited from 5 large academic hospitals including Fujian Medical University Union Hospital, The Affiliated Hospital of Fujian Medical University, Fujian Provincial Cancer Hospital, Fuzhou General Hospital of Nan Jing Military Command, and Fujian Provincial People's Hospital. These centers are highly regarded teaching hospitals and serve all socioeconomic groups from the local populations.

A total of 476 patients were invited to participate in the study, of whom 452 (response rate 95%) completed the procedures (Table 1). The case inclusion criteria were as follows: newly diagnosed primary incident GC cases with endoscopic biopsy or pathological histologic confirmation (ICD-10 code C16); no previous history of a malignant tumor; no previous surgery related to the digestive system; having a stable medical condition as determined by a physician, and a willingness to participate. The healthy control group was randomly sampled from 8 communities. Total of 496 control participants were finally chosen from the population of 135,000 residents, and 465 controls were successfully interviewed (response rate 94%). The eligibility criteria for the controls included: matches with the patients regarding age (within 2 years old), gender, and race; on history of malignancy and digestive disease; no history of serious chronic diseases, such as hypertension or diabetes; and women who were not pregnant and breastfeeding. All participants were of Chinese origin, at least 18 years old, lived in the area for at least 15 years, and psychologically and mentally healthy were able to understand and provide reliable answers to questions, and had undergone standard gastroscopy with *H pylori* tests.

**TABLE 1:**
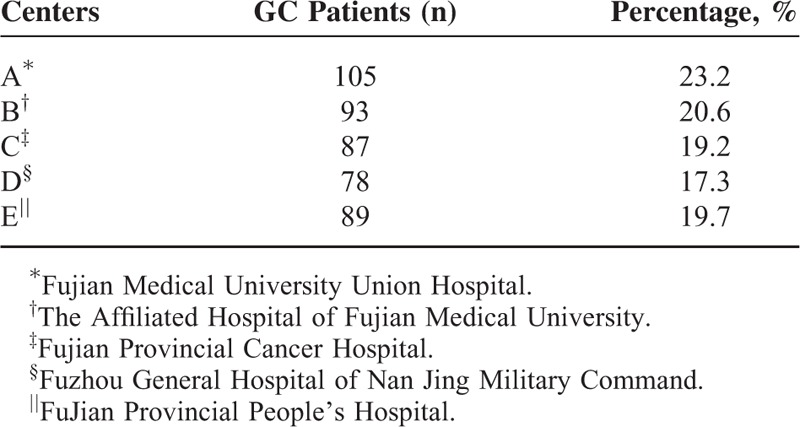
The Number and Percentage of Gastric Cancer (GC) in Different Centers

The ethics committee of Fujian Medical University and each collaborating institution reviewed and approved the study protocol. All participants provided written consent for their information to be collected and used for research, and they had the right to withdraw from the study at any time without prejudice.

### Data Collection

Interviewers administered a unified structured questionnaire during face-to-face interviews. Patients were interviewed at the hospital within 1 week of receiving a diagnosis of GC, and controls were interviewed at community health service centers during the same period. All participants provided information about their lifestyles and behaviors over the last 15 years. The questionnaire consisted of 3 sections including: general demography data (i.e., age, gender, ethnicity, living status, education level, marital status, occupation, local environmental pollution, and body mass index [≤18.0, 18.0–24.9, ≥25, denotes underweight, normal weight, and overweight, using the recommended Chinese standards]);^20^ lifestyle factors (i.e., cigarette smoking [no/yes], alcohol drinking [no/yes], special dietary habit [no/yes], fresh vegetable intake [≥7, 2–6, or ≤1 time/week], fresh fruit intake [≥7, 2–6, or ≤1 time/week], *H pylori* infection [negative/positive], and family cancer history [no/yes]; and information on dinner-to-bed time and post-dinner walk. After finishing these baseline questions, we asked the participants: “Usually, how long is the typical interval between finishing eating dinner and going to bed? (This question was defined as occurring more than 3 days a week or more than 6 months a year).”^13,16^ Then, the participants were asked: “Do you usually take a walk outside after dinner?” The response options were yes or no; the former was defined as an average of 3 times a week or more for at least half of all the weeks in a year for more than 30 minutes each time.^15,21^*H pylori* infection and familial cancer histories were obtained from patients’ medical records and examination reports. These variables were categorized as follows: dinner-to-bed time (t ≥ 4, t = 3–3.9, t = 2–2.9, and t < 1.9 hours), post-dinner walk (walk or nonwalk).

### Variables and Definitions

Each variable was defined based on the literature. Smoking was defined as more than 1 cigarette per day for at least 6 months.^22^ Alcohol drinking was defined as consuming at least 1 drink per week (beer ≥500 mL, wine/liquor ≥200 g) for more than 6 months.^23^ Special dietary habits included eating spicy, fried, smoked, moldy, hot, or picked foods on a daily basis.^15^ A family history of cancer means the first-degree relatives with any type of cancer.^24^ Body mass index = weight (kg) divided by height (m) squared (body height and weight 5 years before interviewed).^25^ Local environmental pollution was assessed as living in a residential area within 5 km of an environmental hazard, such as polluted air or water, etc.^26^

### Quality Control

The study quality control included:

1. Developing the questionnaire based on literatures and consultation with epidemiology and gastrointestinal surgery specialists.
2. Professional training for all investigators.
3. A double-blind research design with regard to both the research hypothesis and objectives.
4. Before the formal interview, 60 patients with GC were randomly selected to complete the same questionnaire twice (within 2–6 weeks). The Spearman coefficient for correlation between the 2 identical questionnaires was 0.76, indicating that the questionnaire was suitable for use in this study; and these 60 patients were not included in the final sample.
5. The investigation was conducted in a quiet environment using plain language.
6. All participants answered the questions personally and each interview took approximately 25 minutes to complete.

### Statistical Analyses

The questionnaire was numbered, and the database was established (via double entry) using Epidata 3.1; a randomly selected sample of 10% was reviewed. The differences between the cases and controls with regard to the demographic characteristics were described using student *t* test or Chi-square test. A conditional logistic regression model was used to compute odds ratio and 95% confidence intervals (95% CIs) to evaluate the associations between the variables and the risk of GC. Based on the risk factors identified in the multivariate logistic regression model, a nomogram was created with *R* using the “rms” packages to visualize the relationships between GC and both dinner-to-bed time and post-dinner walk, which was verified using a calibration plot and bootstrap resampling.^27^ Then, dinner-to-bed time was classified into 2 categories: ≥3 or <3 hours, and a restricted cubic spline model was used to determine the effects of a shorter dinner-to-bed time and post-dinner nonwalk on GC across different age groups (stratified by 10-year intervals), after adjusting for confounding factors.^28^ Finally, a logistic regression model was used to examine the adjusted odds ratio (AOR) of the combine effect of dinner-to-bed time and post-dinner walk. When OR_11_/OR_00_ > (OR_10_/OR_00_) (OR_01_/OR_00_), a multiplicative interaction effect is indicated, and if the 95% CIs of the synergy index (SI) do not equal 1, then the 95% CIs of the relative excess risk of interaction and attributable proportion of interaction do not include 0, which suggests the presence of an additive interaction effect.^29^ All tests were 2-sided and *P*-values <0.05 were considered as significant. The data were analyzed with STATA13.0 (Stata Corp, College Station, TX) and R3.0 (http://www.r-project.org).

## RESULTS

Participant details are presented in Table 2. In all, 917 participants (452 cases, 305 males and 147 females; 465 controls, 307 males and 158 females) were included in our study. All participants were stratified into 6 groups based on age (21–30, 31–40, 41–50, 51–60, 61–70, and 71–85 years old). The average ages of the patients and controls were 60.3 ± 11.3 and 59.89 ± 11.6 years old, respectively. Patients reported significantly dinner-to-bed time <3 hours than controls (*P* < 0.001), and the percentage of post-dinner nonwalk habits was higher in patients than that in controls (80.8% vs 49.7%, *P* < 0.001). There were no statistical differences in demographic characteristics between the 2 groups (*P* < 0.05).

**TABLE 2:**
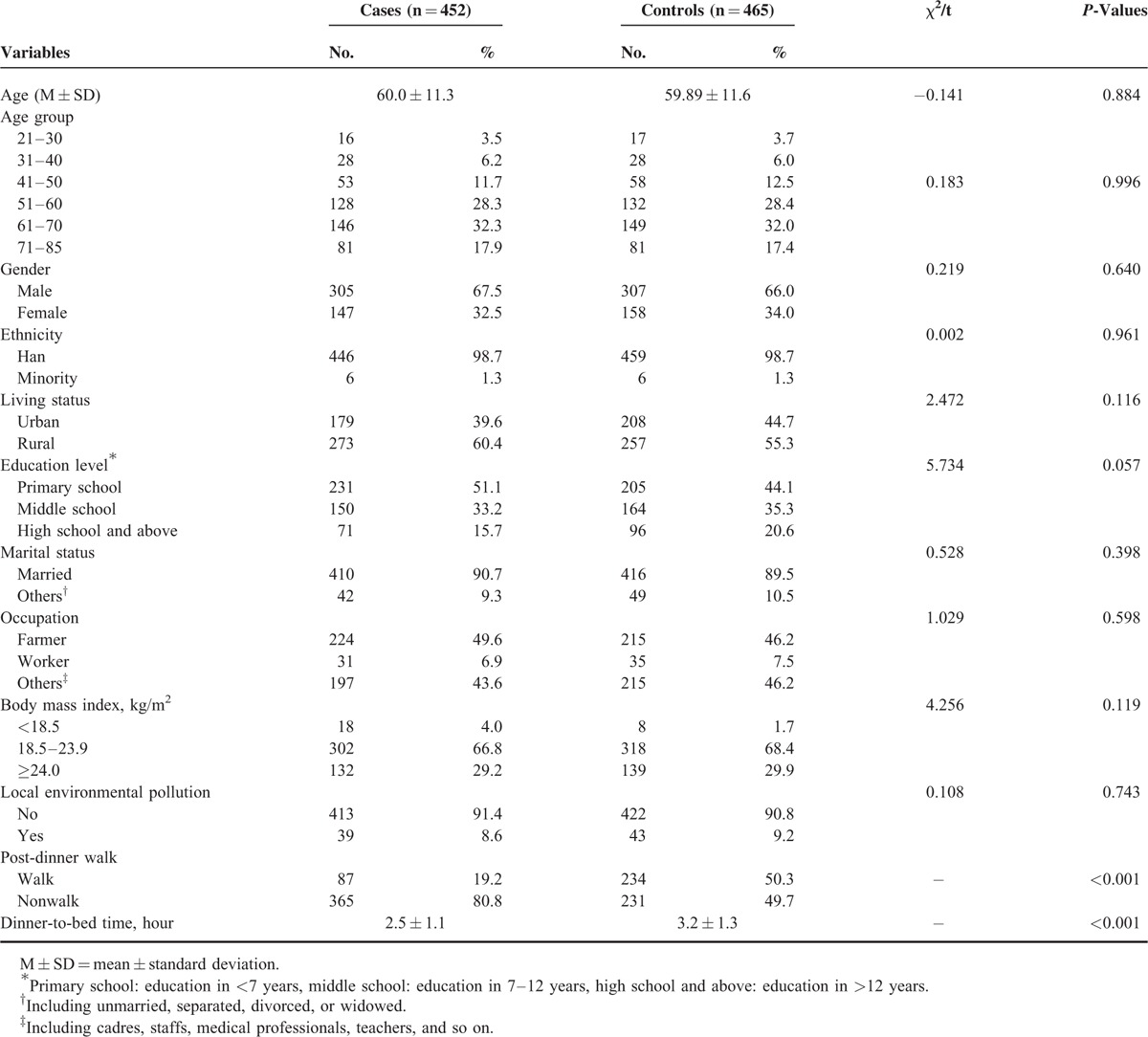
Characteristics of Gastric Cancer Cases and Controls

Table 3 presents the results from the univariate and multivariate analyses between the putative risk factors and GC. When other potential confounders were adjusted, dinner-to-bed time <3 hours was significantly associated with an increased risk of GC, compared with dinner-to-bed time ≥4 hours. Moreover, the shorter the dinner-to-bed time was, the greater was the risk for GC (t = 2–2.9 hours, AOR = 2.712, 95% CIs = 1.755–4.192; t ≤ 1.9 hours, AOR = 4.358, 95% CIs = 2.172–9.524, *P*_trend_ ≤ 0.001), post-dinner nonwalk showed a considerably stronger effect on the risk for GC than post-dinner walk, the AOR was 2.942 (95% CIs = 2.072–4.179).

**TABLE 3:**
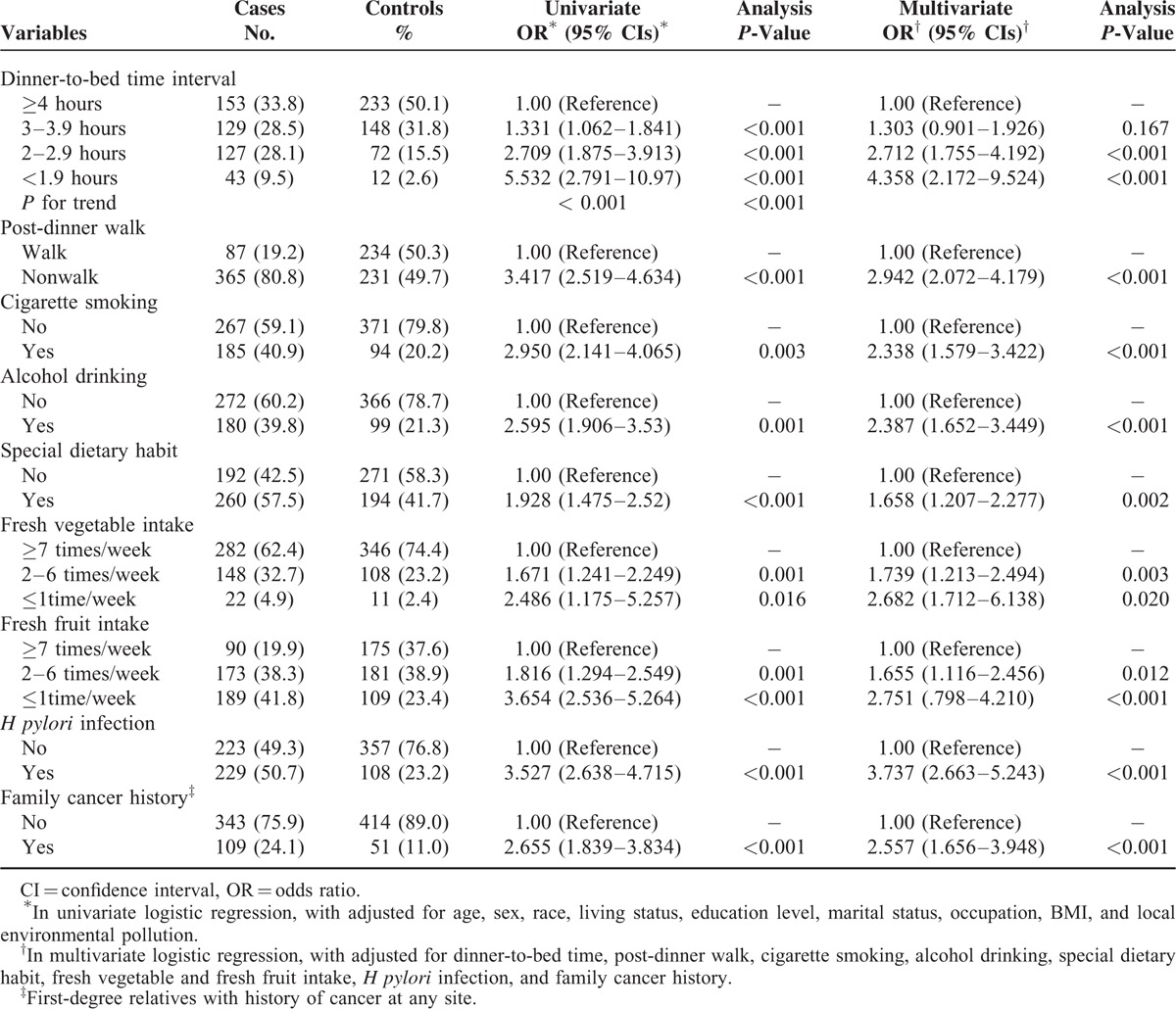
OR (95% CIs) for the Association of Gastric Cancer Risk Among Dinner-to-Bed Time, Post-Dinner Walk, and Other Behavior-Related Factors

**FIGURE 1:**
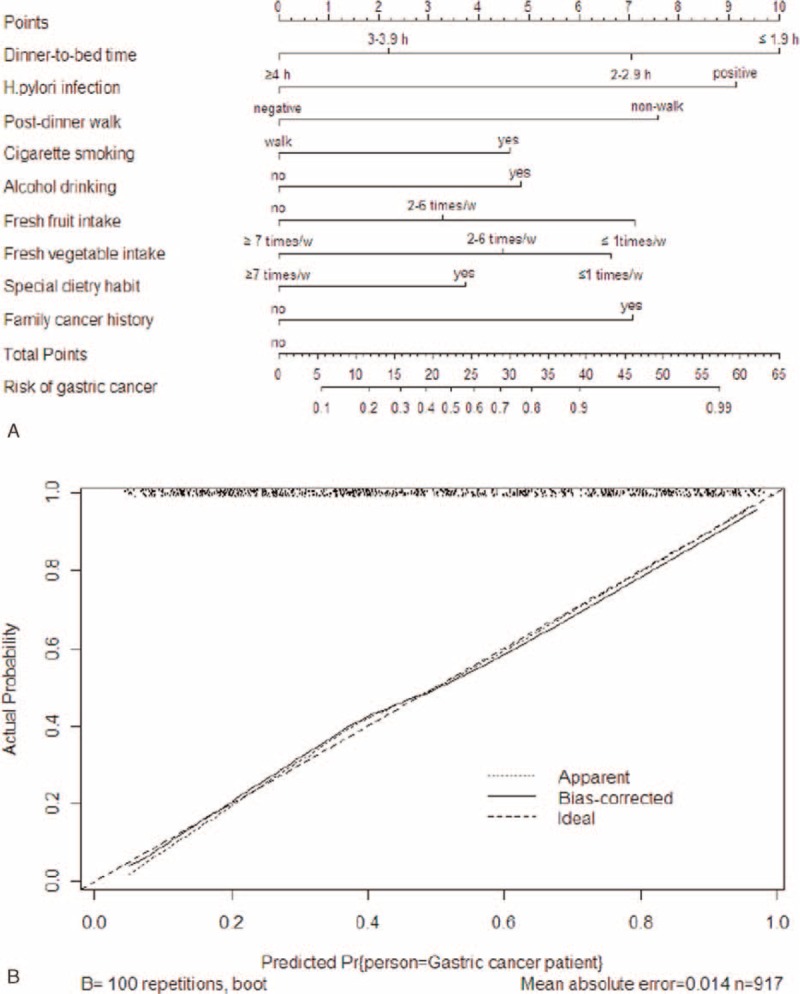
(A) Nomogram with dinner-to-bed time, post-dinner walk, and other risk factors predicted the probability of gastric cancer. Each risk factor corresponds to a specific point by drawing a line straight upward to the Points axis. The sum of the points is located on the total Points axis. (B) Calibration plots for predicting gastric cancer after 200 bootstrap self-sampling internal validation. The C-index was 0.808 (95% confidence intervals [CIs] = 0.618–0.869).

The multivariate analysis revealed that the dinner-to-bed time, post-dinner walk, cigarette smoking, alcohol drinking, special dietary habits, fresh fruit intake, fresh vegetable intake, family history of cancer, and *H pylori* infection were independent risk factors for GC in this study (Table 3). A nomogram was created based on these independent risk variables to examine the risk between the development of GC and both dinner-to-bed time and post-dinner walk. We intuitively found that the risk factor mostly strongly associated with GC was dinner-to-bed time <1.9 hours, which corresponded to the highest integral value in the nomogram, followed by *H pylori* infection and post-dinner nonwalk, with a C-index of 0.808 (95% CIs = 0.618–0.869), which was shown in Figure 1A and B.

**FIGURE 2:**
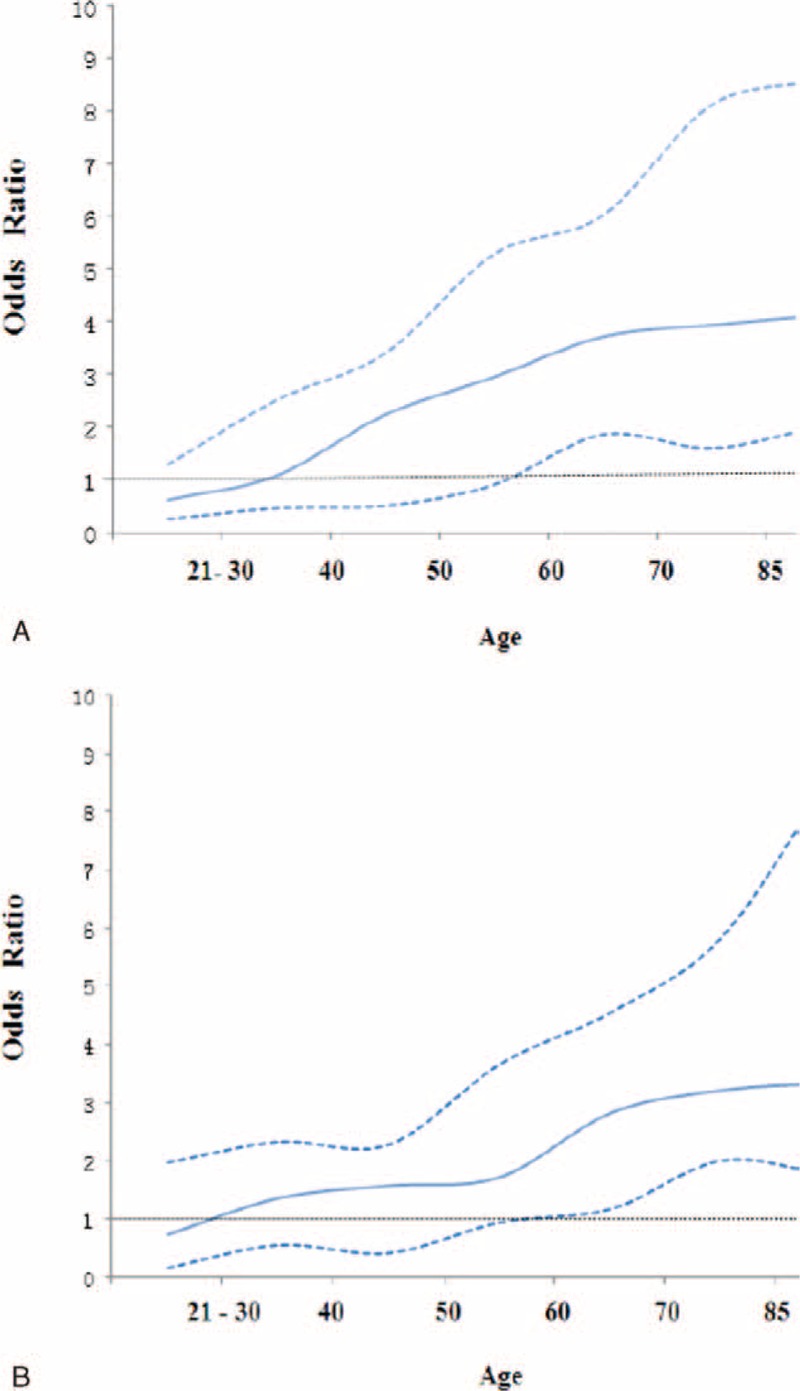
Restricted cubic spline model of the association among dinner-to-bed time <3 hours, post-dinner nonwalk and the risk of GC across different ages. (A) The relationship between dinner-to-bed time <3 hours and GC. (B) The relationship between post-dinner nonwalk and GC. Solid lines and dashed lines represent the estimated OR and its 95% CIs from the spline model, respectively. The OR were adjusted for age, gender, ethnicity, living status, education level, marital status, occupation, BMI, local environmental pollution, dinner-to-bed time, post-dinner walk, cigarette smoking, alcohol drinking, special dietary habit, fresh vegetable, fresh fruit intake, *H pylori* infection, and family cancer history. Dinner-to-bed time ≥3 hours and post-dinner walk was reference. BMI = body mass index, CI = confidence interval, GC = gastric cancer, OR = odds ratio.

The effects of dinner-to-bed time, post-dinner walk, and age on GC were further analyzed using the restricted cubic spline model shown in Figure 2A and B. Dinner-to-bed time ≥3 hours and post-dinner walk were the reference group, respectively. After adjusting for confounders, dinner-to-bed time <3 hours and post-dinner nonwalk were associated with an increased risk for GC as age increased. Specifically, the risk for GC was highest among individuals ≥70 years old; however, no significant effects were observed for people ≤55 years old (95% CIs = 1).

**FIGURE 3:**
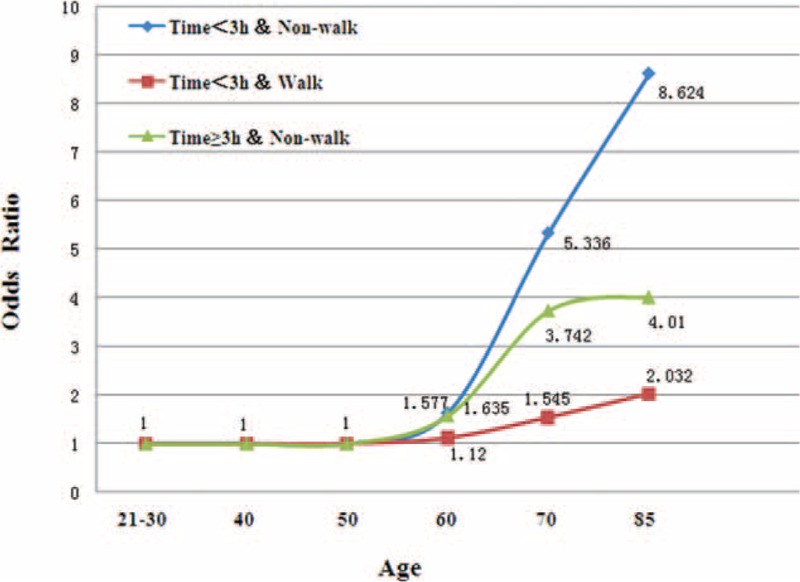
Interactions (OR) between dinner-to-bed time and post-dinner walk with GC at different ages. The interactions were adjusted for age, gender, ethnicity, living status, education level, marital status, occupation, BMI, local environmental pollution, dinner-to-bed time, post-dinner walk, cigarette smoking, alcohol drinking, special dietary habit, fresh vegetable and fresh fruit intake, *H pylori* infection, and family cancer history. Dinner-to-bed time ≥3 hours and post-dinner walk was reference. The combinations of the 2 factors showed greatest risks on GC, particularly at the dinner-to-bed time <3 hours and post-dinner nonwalk. The interaction of the 2 factors was significant among people >55 years old. BMI = body mass index, GC = gastric cancer, OR = odds ratio.

The interaction effects of the dinner-to-bed time and post-dinner walk on the risk for GC are explored in Table 4. The risk for GC was highest when dinner-to-bed time <3 hours co-occurred with post-dinner nonwalk (AOR = 7.401, 95% CIs = 4.523–13.16). Post-dinner walk decreased the risk for GC 6-fold among participants with dinner-to-bed time <3 hours. Furthermore, dinner-to-bed time ≥3 hours reduced the risk for GC by 2.2 times among those who did not take post-dinner walk (95% CIs = 2.201–4.915). The multiplicative interaction between the dinner-to-bed time and post-dinner walk was associated with an AOR of 1.862 (95% CIs = 1.584–3.885) and SI > 1 (AOR = 2.654, 95% CIs = 2.27–3.912), implying that the dinner-to-bed time significant interacted with post-dinner walk (Table 4). In addition, this interaction resulted in an even greater risk for GC among people >55 years old (Figure 3).

**TABLE 4:**
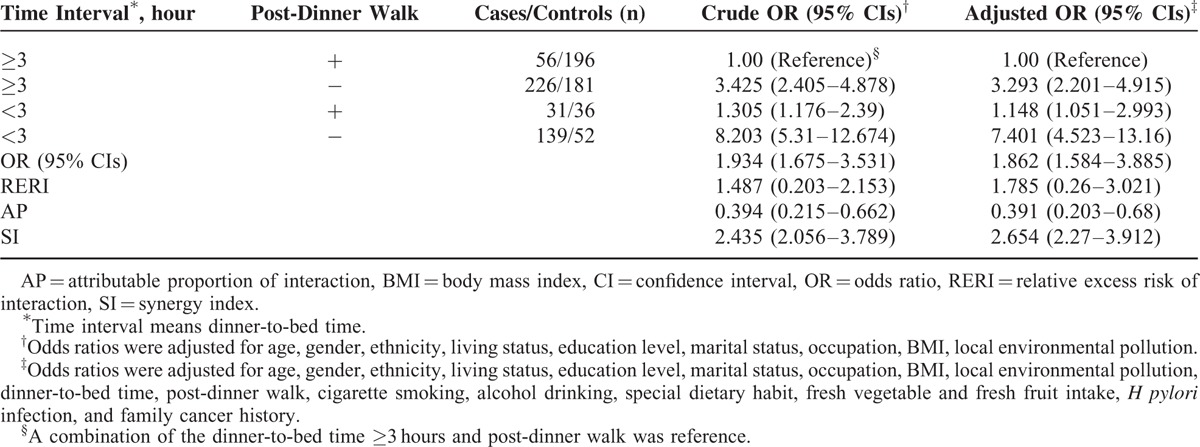
Interactions Between Dinner-to-Bed Time and Post-dinner Walk on Gastric Cancer Risk

## DISCUSSION

GC poses a major threat to global public health, especially in East Asia.^30,31^ Strengthening prevention programs and reducing the incidence of GC is an important issue among scholars. The dinner-to-bed time is a newly defined concept that influences digestive system disease. Dinner-to-bed time <3 hours significantly increase the risk for GERD (AOR = 7.45);^13^ moreover, if reflux symptoms are severe and persist, then they can seriously damage the stomach and esophageal mucosa, and mucosal erosion can eventually result in the development of GCA.^32^ In 2014, Song et al^15,16^ conducted a hospital-based, single center study with a small sample size and found that shorter dinner-to-bed time was positively associated with GCA and ESCC risk. In our study, we found that dinner-to-bed time of <3 hours was strongly associated with an elevated risk for GC. Furthermore, the shorter dinner-to-bed time positively predicted the higher risk of GC (AOR: −2.9 hours = 2.712, −1.9 hours = 4.358). From a biological viewpoint, dinner-to-bed time <3 hours might reduce stomach motility and cause delayed gastric emptying, which resulting in food retention in the stomach. This food is not easy to digest and continues to stimulate the gastric antrum, thereby decreasing the gastric mucosa barrier and encouraging carcinogenicity.^33,34^

The relationship between physical activity and cancer has received intensive investigation recently. Research has suggested that physical activity reduces the risk of many types of cancer, including breast cancer, colorectal cancer, ovarian cancer, and bladder cancer;^35,36^ however, the effect of physical activity on GC remains controversial.^18,37^ Schmid and Leitzmann^18^ recently published systematically reviewed observational studies found that physical activity was not related to the incidence of GC. By contrast, several meta-analyses have suggested that physical activity has a protective effect against GC, regardless of whether the activity performed is occupational or recreational.^38,39^ Our study found a nearly 3-fold elevated risk of contracting GC among people who did not take post-dinner walk (AOR = 2.942) compared with those who did. The finding suggests that post-dinner walk is an independent protective factor for GC. As a systematic exercise, post-dinner walk stimulates parasympathetic nervous excitement, promotes gastrointestinal motility, and accelerates gastric emptying, thereby promoting food digestion.^40^ Additionally, Bradley et al^41^ demonstrated that regular physical activity not only decreased the concentrations of inflammatory factors, but also increased the antiinflammatory cytokines that reduce the incidence of cancer.^42^ In our opinion, post-dinner walk produces beneficial movement and is conductive to mental relaxation, which enhances the ability of the body's immune system to fight disease.

We also examined the evidence for a 2-way interaction between the dinner-to-bed time and post-dinner walk on GC. We found that dinner-to-bed time interacted with post-dinner walk (AOR = 1.862, SI > 1). Interactions among factors have been extensively studied regarding the etiology of cancer. An earlier study from India reported a significant interaction between smoking and drinking on the risk of oral cancer.^43^ In their case–control study, Wang et al^44^ found that *H pylori* infection increased the risk for GCA through interactions with lifestyle. A recent population-based, case–control study in China reported that salted meat combined with alcohol drinking and smoking at least 10 times increased the risk for ESCC compared with salted meat consumption alone.^25^ In our study, which was adjusted for confounders, participants who had dinner-to-bed time <3 hours and post-dinner nonwalk were at a 7.4-fold increased risk of developing GC (95% CIs = 4.532–13.16) compared with those with dinner-to-bed time ≥4 hours and post-dinner walk. However, those with dinner-to-bed time <3 hours who took post-dinner walk were 6.4 times less likely to develop GC (AOR = 1.148). This result implies that post-dinner walk can mitigate the effect of dinner-to-bed time <3 hours on the risk for GC, which is consistent with the findings of other studies on GCA and ESCC.^15,16^ The precise mechanisms of the associations among GC, dinner-to-bed time <3 hours, and post-dinner walk are unknown.

Another major finding from our study is that dinner-to-bed time, post-dinner walk, and their joint effect increase the risk for GC with age. Bodily functioning gradually declines with age, and gastric dilatation and the secretion of digestive enzymes decrease in the elderly, which directly decrease of digestive functioning.^45^ Moreover, shorter dinner-to-bed time with post-dinner nonwalk might delay the digestion of food in the stomach, increasing digestion burden and thereby enhancing the risk for GC among the elderly. Thus, the synergic effects of shorter dinner-to-bed time and post-dinner nonwalk significantly increased the risk for GC with age more than single-factor effect. Regardless of their alone or interaction, however, dinner-to-bed time and post-dinner were negatively associated with the occurrence of GC in people ≤55 years old, perhaps because this disease is a long-term and gradual process.

In 2013, 8.2 million people died of cancer making it the most common cause of death wordwide.^1^ The World Health Organization noted that at least one-third of all cancers can be prevented, and only targeted preventive measures can greatly reduce the morbidity and mortality associated with this disease.^46^ A meta-analysis of international cancer research demonstrated that 34.7% of all cancer-related deaths are attributable to 9 types of detrimental behaviors and environmental factors.^47^ Therefore, the identification of environmental and lifestyle risk factors as well as the application of corresponding primary prevention measures might greatly reduce the burden of cancer. Our study discovered a new interaction effect between the dinner-to-bed time and post-dinner walk that might affect GC development. In particular, attention should be paid to people who are >55 years old. The behavior of dinner-to-bed time exists in our daily lives, but it is not as addictive as smoking and drinking and can be relatively easy to correct and intervene. Consequently, it is important to disseminate the knowledge of going to bed more than 3 hours after dinner and taking a post-dinner walk among the general population. Although, our study found that dinner-to-bed time and post-dinner walk only affected the risk for GC among people >55 years old, one should not assume that these factors unimportant among people ≤55 years old. So, it is necessary to develop healthy lifestyles, regardless of age.

To the best of our knowledge, the present study is the first to investigate the interaction effect between dinner-to-bed time and post-dinner walk on the risk for GC. This study has some potential limitations. As with all retrospective case–control studies, recall and selection biases cannot be fully avoided; although we adopted strict quality control schemes. The healthy control group might have a better sense of their own healthcare than patients with GC. In addition, although the present sample represented the largest in the field, it remained relatively small for specific subgroup analyses. Nevertheless, several strengths of our study should be noted. First, on the basis of multicenter and population-based case–control study design that has reasonable representative sample of cases and healthy controls. Second, we conducted our investigation within 1 week after the diagnosis of GC, and every item on the questionnaire was clearly defined to reduce recall errors. Third, many widely established risk and protective factors of GC were included in this study, specifically *H pylori* as a first degree carcinogen for GC,^48^ which minimized the potential influence of confounders on the results of the study. Fourth, the case and control groups had balanced baseline data, guaranteeing the authenticity and comparability of the research results. Lastly, although this study was retrospective, it might serve as a reference for future prospective studies.

## CONCLUSIONS

In summary, we found that dinner-to-bed time <3 hours and post-dinner nonwalk are independent risk factors for GC, and the synergistic effect between these factors significantly increases the risk for GC, especially among people >55 years old. Therefore, actively advocating for longer dinner-to-bed time and regular post-dinner walk might help to prevent GC. Large-scale prospective cohort studies are needed to further explore these associations.
